# Oleanolic Acid Decreases IL-1*β*-Induced Activation of Fibroblast-Like Synoviocytes via the SIRT3-NF-*κ*B Axis in Osteoarthritis

**DOI:** 10.1155/2020/7517219

**Published:** 2020-09-28

**Authors:** Jiapeng Bao, Weifeng Yan, Kai Xu, Mengyao Chen, Zhonggai Chen, Jisheng Ran, Yan Xiong, Lidong Wu

**Affiliations:** ^1^Department of Orthopedics Surgery, The Second Affiliated Hospital, Zhejiang University School of Medicine, Hangzhou, China; ^2^Department of Orthopedics, China Coast Guard Hospital of the People's Armed Police Force, Jiaxing, China; ^3^Department of Medical Oncology, The Second Affiliated Hospital, Zhejiang University School of Medicine, Hangzhou, China

## Abstract

Synovial inflammation is a major pathological feature of osteoarthritis (OA), which is a chronic degenerative joint disease. Fibroblast-like synoviocytes (FLS), localized in the synovial membrane, are specialized secretory cells. During OA synovitis, FLS produce chemokines and cytokines that stimulate chondrocytes to secrete inflammatory cytokines and activate matrix metalloproteinases (MMPs) in FLS. Recent studies have demonstrated that sirtuin 3 (SIRT3) performs as a key regulator in maintaining mitochondrial homeostasis in OA. This study aims at ascertaining whether SIRT3 is involved in OA synovitis. The overexpression (OE) and knockdown (KD) of SIRT3 are established by short hairpin RNA (shRNA) and recombinant plasmid in human FLS. The anti-inflammatory effect of SIRT3 underlying in oleanolic acid- (OLA-) prevented interleukin-1*β*- (IL-1*β*-) induced FLS dysfunction is then evaluated in vitro. Additionally, the molecular mechanisms of SIRT3 are assessed, and the interaction between SIRT3 and NF-*κ*B is investigated. The data suggested that SIRT3 can be detected in human synovial tissues during OA, and OLA could elevate SIRT3 expression. OE-SIRT3 and OLA exhibited equal authenticity to repress inflammation and reverse oxidative stress changes in IL-1*β*-induced human FLS dysfunction. KD-SIRT3 was found to exacerbate inflammation and oxidative stress changes in human FLS. Furthermore, it was found that SIRT3 could directly bind with NF-*κ*B, resulting in the suppression of NF-*κ*B activation induced by IL-1*β* in human FLS, which then repressed synovial inflammation in OA. In general, the activation of SIRT3 by OLA inhibited synovial inflammation by suppressing the NF-*κ*B signal pathway in FLS, and this suggested that SIRT3 is a potential target for OA synovitis therapy.

## 1. Background

Osteoarthritis (OA) is the most common joint degenerative disease, characterized by cartilage degradation, underlying bone remodeling, and synovial inflammation [1]. The etiology of OA is complex and involve genomics, proteomics, and cytomics. Many studies have explored the development mechanism of OA, including low-grade inflammation research, epigenetic research, DNA methylation, microRNA, long noncoding RNA, and circRNA research [2]. Growing evidence has demonstrated that synovial inflammation, which is clinically described as synovitis, accelerates the development of OA [3]. Synovial fluid (SF) biomarkers, such as CD14, CD163, and SF elastase, can predict knee OA progression. Recent studies have shown that the SF biomarker, VEGF, a signal protein of angiogenesis, is associated with structural and symptom severity of OA, which increased MMP-9, MMP-13, and the initiation of OA [4]. It is well documented that synovium can produce synovial fluid and nutrition for joint homeostasis, and its function is essential for physiological behaviors [5]. Fibroblast-like synoviocytes (FLS), the crucial resident cells in the synovium, constitute up to 75% of all cells in the synovium and maintain the synovial tissue extracellular matrix and synovial fluid homeostasis [6]. During OA, FLS proliferate rapidly and attach to the articular cartilage. FLS also produce inflammatory cytokines, such as cyclooxygenase-2 (COX2) and prostaglandin 2(PGE2), and activate matrix metalloproteinases (MMPs) [7, 8]. Furthermore, in OA synovitis, FLS adhere to chondrocytes and the latter secrete inflammation mediators and activate MMPs, resulting in the acceleration of OA progression [9, 10]. However, the mechanisms underlying this molecular chemical reaction still remain unknown.

Mitochondria, a membrane-enclosed organelle, preforms a crucial role in cell metabolism. Some studies have shown that mitochondria dysfunction is a hallmark of aging and contributes to the pathophysiological process of OA [11]. In addition, some studies have demonstrated that protein acetylation may induce mitochondria dysfunction and chondrocyte inflammation. SIRT3, as a major mitochondrial protein deacetylase localized in mitochondria, is a key regulator in OA [12]. Our previous study confirmed that the overexpression SIRT3 could inhibit inflammation induced by IL-1*β* in rat chondrocytes. In vivo, it was demonstrated that overexpression of SIRT3 alleviated cartilage degeneration. However, to the best of our knowledge, nothing has been reported regarding SIRT3 in synovial inflammation.

OLA is a triterpene that has been isolated from *Fructus Ligustri Lucidi*, the fruit of *Ligustrum lucidum* [13]. Various studies have demonstrated that OLA has anti-inflammatory effects. Some studies have shown that OLA can mitigate IL-1*β*-induced chondrocyte dysfunction [13–15].

In this study, the primary aim is to determine whether OLA can alleviate IL-1*β*-induced FLS inflammation and dysfunction and whether OLA regulates SIRT3. Furthermore, the function of SIRT3 on inflammation and the molecular mechanisms in human FLS are also investigated.

## 2. Materials and Methods

### 2.1. Reagents

Oleanolic acid (OLA) was purchased from Sigma, USA, CAS508-02-1. Recombinant human IL-1*β* was purchased from R&D Systems, Abingdon, UK. The OLA was dissolved in dimethyl sulfoxide (DMSO), and the IL-1*β* was dissolved in phosphate buffered saline (PBS). Dulbecco's modified Eagle's medium (DMEM), penicillin/streptomycin, fetal bovine serum (FBS), and 0.25% trypsin were purchased from Gibco BRL, Grand Island, NY, USA. The collagenase I and DMSO were purchased from Sigma-Aldrich, St. Louis, MO, USA.

### 2.2. Cell Culture

All of the synovial tissues were obtained from eight patients (six females, mean age 67 yrs, and two males, mean age 63 yrs) who suffered from OA of the knee and underwent total knee arthroplasty (TKA). All of the patients signed an informed consent form that was approved by the Ethics Committee of the Second Affiliated Hospital, School of Medicine, Zhejiang University, Hangzhou, China. The synovial membranes were minced and incubated with 0.1% type I collagenase on a horizontal shaker at 37°C for 4 h. The cell suspension was seeded and grown in DMEM supplemented with 10% FBS, 100 units/ml of penicillin, and 100 ug/ml streptomycin in a 37°C and 5% CO_2_ incubator. These cells were considered passage0 (P0). After cell passaging at a ratio of 1 : 3, the P3 cells were primarily FLS. P3–P5 FLS were used for the assays.

### 2.3. Cell Viability Assay

The cytotoxicity of OLA on FLS was determined using the cell counting kit-8 (CCK-8, Dojindo, Kumamoto, Japan). The FLS were seeded in 96-well plates at a density of 1 × 10^4^ per well. Then, these FLS were treated with OLA at different concentrations (0, 1, 10, 20, 50, and 100 *μ*M) for 24, 48, and 72 h separately. Cell viability was determined using CCK-8 assays according to the manufacturer's instructions. All assays were performed in triplicate.

### 2.4. Immunohistochemical Stain

The synovial tissues were obtained from eight patients (five females, mean age 65 yrs, and three males, mean age 62 yrs) who suffered from OA of the knee and underwent total knee arthroplasty. The paraffin blocks were then cut into 5 mm sections. These sections were blocked with hydrogen peroxide for 20 min prior to pepsin treatment. Then, these sections were blocked with 5% BSA (Sigma-Aldrich, St. Louis, MO, USA) for 1 h at room temperature before being incubated with primary antibody against SIRT3(Abcam, #ab264041) at 4°C overnight. After that, peroxidase-linked secondary antibody was used for 1 h at room temperature incubation, and 3,30-diaminobenzidine was used as a chromogenic agent. The results were viewed using optical microscopy.

### 2.5. Knocking down of SIRT3 (SIRT3-KD) by Lentiviral Short Hairpin RNA (shRNA)

The SIRT3 expression silencing using small hairpin RNA (shRNA) was designed and synthesized by Genechem Co. Ltd. (Shanghai, China). The recombinant lentivirus of shRNA targeting SIRT3 (SIRT3-RNAi-lentivirus) and the control lentivirus (GFP-lentivirus) were generated for the SIRT3-knockdown experiment. The sequences of shRNA were as follows: shRNA1 (5′-CCCTGGCCGTGTGTTTGATAT-3′), shRNA2 (5′-CCGTAAAGGTGTGGCTATTAA-3′), and shRNA3 (5′-ATTCTGCAGCAGATTGAATTA-3′). Briefly, pLKO.1 was constructed as the lentivirus transfer vector, which contained a puromycin resistant gene. According to the three interference sites above, shRNA-pLKO-1 vectors were constructed. 293T cells were cotransfected with shRNA-pLKO-1, psPAX2, and pMD2.G vectors, which were purchased from Genechem Co. Ltd. (Shanghai, China). During transfection, transfected cells were screened using puromycin, and lentiviral particles were collected. The lentiviral-shRNA was transfected into the FLS, and the knockdown efficiency of shRNA was evaluated using qRT-PCR.

### 2.6. Overexpression of SIRT3 (SIRT3-OE) by a Recombinant Plasmid

Target genes (SIRT3, NF-*κ*B) were obtained from human FLS using PCR. The expression plasmids, pcDNA6-Flag (BamHI/XhoI) and pcDNA6-Myc (BamHI/XhoI), were obtained from Invitrogen (Groningen, The Netherlands). Transfection was performed using GeneArt® Seamless Cloning and Assembly Enzyme Mix (Invitrogen, #A14606) for 24 h according to the manufacturer's instructions. The transfection efficiency was tested using the Sanger sequencing (Sangon Biotech, Shanghai, China).

### 2.7. ELISA Assay

The FLS were divided into six groups: a normal group, IL-1*β* group, SIRT3-OE group, IL-1*β*+SIRT3-OE group, IL-1*β*+SIRT3-KD group, and IL-1*β*+oleanolic acid group. SIRT3 was an overexpression or knockdown first in the FLS. Next, the FLS were treated with 100 *μ*M of OLA for 1 h and then with 10 ng/ml of IL-1*β* for a further 24 h in the IL-1+oleanolic acid group. In the IL-1*β* group, IL-1*β*+SIRT3-OE group, and IL-1*β*+SIRT3-KD group, the FLS were treated with isovolumetric DMSO as a control for 1 h then with 10 ng/ml of IL-1*β* for a further 24 h. After treatment, the FLS were collected. Prostaglandin E_2_ (PGE_2_) and cyclooxygenase-2 (COX2) levels in the FLS were measured using a PGE_2_ high-sensitivity ELISA Kit (Abcam, #ab133055) and a COX2 high-sensitivity ELISA Kit (Abcam, #ab267646) according to the manufacturers' instructions. The sensitivity and measuring range of the COX2 ELISA Kit were 1.2 ng/ml and 1.229 ng/ml–300 ng/ml, respectively. The sensitivity and measuring range of the PGE_2_ ELISA Kit were 8.26 pg/ml and 7.8 pg/ml–1000 pg/ml, respectively. All assays were performed in triplicate.

### 2.8. Quantitative Real-Time PCR

The FLS were cultured and pretreated into different groups. A TRIzol® Plus RNA Purification Kit (Invitrogen, Carlsbad, CA, USA) was used to extract the total RNA from the FLS on ice. The total RNA was quantified according to the absorbance at a 260 nm (A260)/A280 ratio by DeNovix. Then, the RNA was reversely transcribed into cDNA using PrimeScript™ RT Master Mix (Takara). The reaction was conducted at 37°C for 15 min followed by 5 min at 85°C. Then, the cDNA samples were replicated using SYBR® Premix Ex Taq™ II (Takara) by ABI StepOnePlus System. Each 10 *μ*l sample contained 5 *μ*l of SYBR® Premix Ex Taq™ II, 0.4 *μ*l of each forward and reverse primer, 1 *μ*l of cDNA (10 ng), and 3.2 *μ*l of ddH_2_O. The reaction conditions were as follows: denaturation, 95°C × 30 sec, followed by 40 cycles of 95°C × 15 sec→60°C × 32 sec→72°C × 1 min→72°C × 5 min. The primers used are shown in Table 1, and the expressions of ADANTS4, INOS, and MMP3 SIRT3 were detected. GAPDH was used as an endogenous control. The formula 2^-*ΔΔ*Ct^ was used to analyze the gene expression data. All assays were performed in triplicate.

### 2.9. Western Blot Analysis

Total FLS proteins were extracted using the RIPA Lysis Buffer containing protease and phosphatase inhibitors for 30 min. Then, the samples were quantified using a BCA Protein Assay kit (Beyotime Biotechnology, Shanghai, China) and then denatured at 100°C for 10 min. They were then separated using 10% sodium dodecyl sulfate- (SDS-) polyacrylamide gels and transferred into nitrocellulose membranes. The membranes were blocked using 5% BSA for 1 h. According to the different protein molecular weights, the membranes were cut into different membranes. These membranes were incubated with different primary antibodies against GAPDH (Abcam, #ab181602), SIRT3 (Abcam, #ab118334), NF-*κ*B p65 (CST, #8242), p-NF-*κ*B p65 (Ser536) (CST, #3033), NF-*κ*B p65 (Acetyl K310) (Abcam, #ab19870), anti-FLAG Tag (Sigma, #SAB430113), and anti-c-Myc Tag (Sigma, #SAB4301136) at 4°C overnight. GAPDH was used as the endogenous control. All antibodies were used at a 1 : 1000 dilution. After being washed three times with TBS-T, the membranes were incubated with secondary antibodies for 1 h. After being washed another three times, the protein bands were luminesced using the Pierce™ ECL Western blotting substrate and were detected with the Bio-Rad ChemiDoc System. All assays were performed in triplicate.

### 2.10. Intracellular Reactive Oxygen Species Analysis

After the different treatments as shown above, the intracellular reactive oxygen species (ROS) in the FLS were detected using the Reactive Oxygen Species Assay Kit (Beyotime, China). The oxidative conversion of the cell permeable 2',7'-dichlorofluorescein diacetate (DCFH-DA) was oxidized into fluorescent dichlorofluorescein (DCF) upon reaction with hydroxyl radical, hydrogen peroxide, or peroxynitrite. The cells were incubated with DCFH-DA at 37°C for 30 min in the dark. Then, all of the samples were washed twice with PBS, and the fluorescence intensity of the intracellular ROS were viewed using a fluorescence microscope and measured using ImageJ.

### 2.11. Coimmunoprecipitation (CoIP) Assay

The FLS were lysed in an immunoprecipitation lysis buffer containing a protease inhibitor. After centrifugation, the collected supernatant was incubated with magnetic beads (Sigma, M8823) at 4°C overnight. Then, the beads were rinsed, and the bound proteins were eluted and then evaluated using a Western blot assay.

### 2.12. Statistical Analysis

All the data are presented as means ± SDs. A one-way analysis of variance (ANOVA) with Tukey's post hoc test was used for multiple comparisons. A value of *p* < 0.05 was considered to indicate a significant difference.

## 3. Results

### 3.1. OLA Increases the Proliferation Activity of Human FLS and the Expression of SIRT3

**Figure 1:**
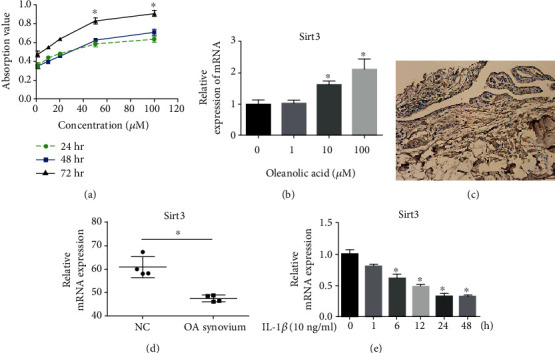
The effect of OLA on human FLS and the expression of SIRT3. (a) Human FLS were treated with increasing concentrations of OLA (0, 1, 10, 20, 50, and 100 *μ*M) for diffident time periods (24 h, 28 h, and 72 h). The effects of OLA on human FLS viability were evaluated via the CCK-8 assay. (b) After treatment with different concentrations of OLA for 24 h, the SIRT3 expression in human FLS was detected using RT-PCR. (c) An immunohistochemical stain was performed to evaluate the expression of SIRT3 in the OA synovium. (d) The mRNA expression of SIRT3 from donors between normal synovial tissue and from the OA synovium. Normal group *n* = 4 (4 females, mean age 45 yrs); OA synovium group *n* = 4 (4 females, mean age 63 yrs). (e) The mRNA expression of SIRT3 decreased by IL-1*β* in FLS. FLS were treated with 10 ng/ml of IL-1*β* for different time periods (0, 1, 6, 12, 24, and 48 h). The expression of SIRT3 significantly decreased at 6, 12, 24, and 48 h compared with 0 h. The values are expressed as means ± standard deviations (SD), ∗*p* < 0.05. Scale bar = 100 *μ*m.

The effects of OLA on human FLS viability was determined using the CCK-8 assay. The results demonstrated that the absorption value of A450 increased with increasing concentrations of OLA at 24 h, 48 h, and 72 h (Figure 1(a)). Compared with OLA concentrations of 0, 1, 10, and 20 *μ*M, the absorption of A450 was significantly increased with OLA concentrations of 50 and 100 *μ*M (*p* < 0.01). The result indicated that OLA could increase the proliferation activity of FLS. According to the CCK-8 assay result and previous studies, 100 *μ*M of OLA was used for the in vitro experiment [14]. Recently, some studies have found that mitochondrial dysfunction was involved in synovitis [16]. SIRT3 is located in the mitochondria and regulates mitochondrial function. This protein was focused on in this study, and it was found that OLA significantly increased the expression of SIRT3 in FLS according to the result of the RT-PCR (Figure 1(b)). It was concluded that OLA increased SIRT3, which increased the proliferation activity of the FLS. In order to explore the function of SIRT3 in synovial tissues, an immunohistochemical for SIRT3 was used, and it was found that SIRT3 was expressed in the synovial membrane of OA (Figure 1(c)). Interestingly, according to the RT-PCR results, it was found that the mRNA expression of SIRT3 decreased in the OA synovium when compared with normal synovial tissues (Figure 1(d)). Then, it was attempted to ascertain whether or not the expression of SIRT3 decreased due to the inflammatory response in FLS. The FLS were treated with 10 ng/ml of IL-1*β* for different times (0, 1, 6, 12, 24, and 48 h). The result of the RT-PCR showed that the SIRT3 expression significantly decreased at 6, 12, 24, and 48 h when compared with 0 h (Figure 1(e)). These results showed that SIRT3 was associated with synovitis, and OLA may affect FLS via SIRT3 regulation.

### 3.2. SIRT3 Contributes to the Attenuation of IL-1*β*-Induced Inflammation in Human FLS

**Figure 2:**
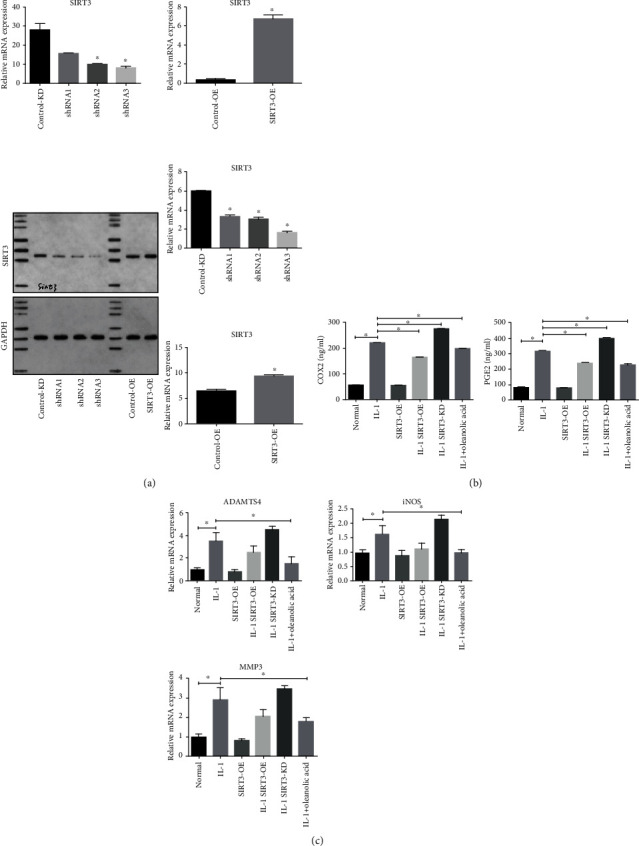
Effect of SIRT3 on IL-1*β*-induced inflammatory genes and MMPs expression in human FLS. FLS were pretreated with SIRT3-OE or SIRT3-KD and then incubated with or without IL-1*β* (10 ng/ml) for 24 h. (a) The efficiency of SIRT3-KD and SIRT3-OE was measured using qRT-PCR and Western blot. (b) The expressions of COX2 and PGE2 were measured using an ELISA assay. (c) The expressions of ADAMTS4, iNOS6, and MMP3 were measured using RT-PCR. The values are expressed as means ± standard deviations (SD), ∗*p* < 0.05.

The RNA interference approach was performed with 3 shRNA to knockdown the expression of SIRT3, and the knockdown efficiency was measured using quantitative real-time PCR and Western blot. The results showed that shSIRT3 had th4e highest knockdown efficiency, and it was selected for the rest of experiments (Figure 2(a)). A recombinant plasmid of SIRT3 was used in overexpression experiments, and the efficiency was also measured using quantitative real-time PCR and Western blot. The results showed that the recombinant plasmid of SIRT3 increased SIRE3 expression significantly (Figure 2(a)). In order to ascertain whether SIRT3 was involved in OA synovitis, an ELISA assay (Figure 2(b)) and RT-PCR (Figure 2(c)) were performed to measure the expressions of COX2, PGE2, ADAMTS4, iNOS6, and MMP3 in IL-1*β*-induced FLS. The results showed that IL-1*β* could significantly upregulate the expression of inflammatory genes (COX2, PGE2, ADAMTS4, and iNOS6) and the matrix-degrading gene (MMP3) in human FLS. Overexpression of SIRT3 (SIRT3-OE) had no effect on the expression of COX2, PGE2, ADAMTS4, iNOS6, and MMP3 in normal human FLS. However, it significantly decreased the IL-1*β*-induced upregulation of COX2, PGE2, ADAMTS4, iNOS6, and MMP3 in human FLS, such as the anti-inflammatory effect of OLA. In contrast, the knockdown the expression of SIRT3 (SIRT3-KD) significantly increased the IL-1 *β*-induced upregulation of inflammatory markers. These data suggest that OLA may contribute to the attenuation of IL-1*β*-induced inflammation via SIRT3 regulation in human FLS.

### 3.3. SIRT3 Decreased IL-1*β*-Induced Intracellular Levels of ROS in Human FLS

**Figure 3:**
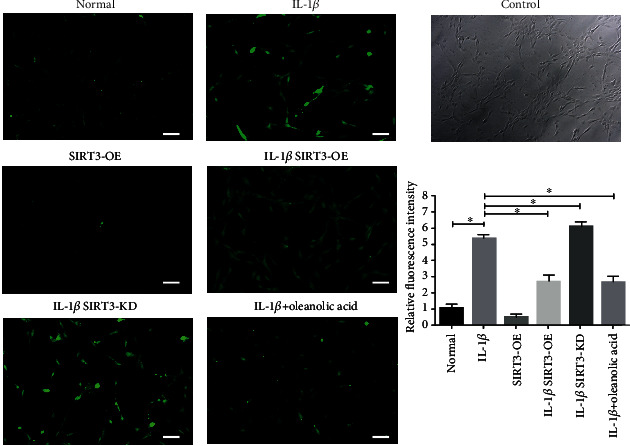
The effects of SIRT3 and OLA on IL-1*β*-induced intracellular levels of ROS in human FLS. FLS were pretreated with SIRT3-OE or SIRT3-KD and then incubated with or without IL-1*β* (10 ng/ml) for 24 h. The ROS were measured and viewed using a fluorescence microscope, and the quantitative analysis is listed. The values are expressed as means ± standard deviations (SD), ∗*p* < 0.05.

The effects of SIRT3 and OLA on ROS were viewed by fluorescence microscope and measured by ImageJ (Figure 3). The overexpression of SIRT3 (SIRT3-OE) significantly decreased the IL-1*β*-induced upregulation of the intracellular level of ROS in human FLS, such as the effect of OLA. In contrast, the knockdown the expression of SIRT3 (SIRT3-KD) significantly increased the IL-1 *β*-induced upregulation of ROS. These data suggest that OLA decreased the IL-1*β*-induced intracellular level of ROS via SIRT3 regulation in human FLS.

### 3.4. SIRT3 Inhibited Phosphorylation and Acetylation of NF-*κ*B

**Figure 4:**
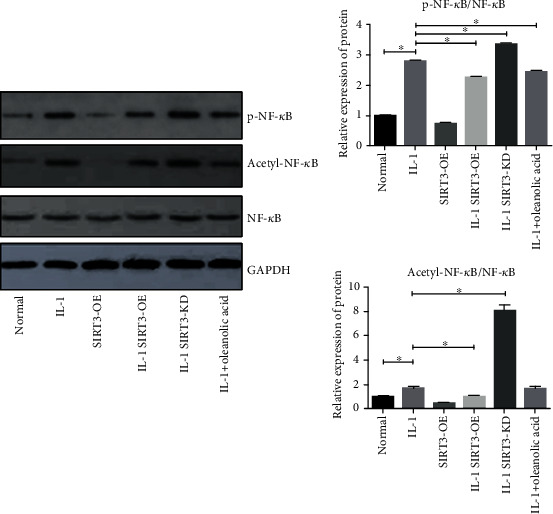
Effect of SIRT3 on the IL-1*β*-induced NF-*κ*B signal activation in human FLS. FLS were pretreated with SIRT3-OE or SIRT3-KD and then incubated with or without IL-1*β* (10 ng/ml) for 30 min. A Western blot analysis and quantitative analysis of p-NF-*κ*B/NF-*κ*B and acetyl-NF-*κ*B/NF-*κ*B in human FLS. The values are expressed as means ± standard deviations (SD), ∗*p* < 0.05.

The effects of OLA on the NF-*κ*B signal pathway were also investigated. The results showed that in the IL-1*β*-activated NF-*κ*B signal pathway, OLA significantly increased the elevation of the p-NF-*κ*B/NF-*κ*B and acetyl-NF-*κ*B/NF-*κ*B ratio. OLA reduced the elevation of the p-NF-*κ*B/NF-*κ*B and acetyl-NF-*κ*B/NF-*κ*B ratio induced by IL-1*β* stimulation (Figure 4). Recent studies have shown that SIRT3 binds NF-*κ*B. However, there are few studies that have investigated this mechanism in synovitis. The NF-*κ*B signal pathway was investigated in this study to explore the underlying mechanism of SIRT3 in synovitis. The effect of SIRT3 on the IL-1*β*-induced NF-*κ*B signal pathway activation was measured in human FLS using a Western blot assay (Figure 4). The results demonstrated that overexpression of SIRT3 significantly reduced the elevation of the p-NF-*κ*B/NF-*κ*B and acetyl-NF-*κ*B/NF-*κ*B ratio induced by IL-1*β* stimulation in human FLS, such as the effect of OLA. The knockdown of SIRT3 significantly increased the IL-1*β*-induced upregulation phosphorylation and acetylation of NF-*κ*B. These results showed that SIRT3 significantly inhibited the IL-1*β*-induced upregulation phosphorylation and acetylation of NF-*κ*B. These results demonstrated that OLA effectively inhibited IL-1*β*-induced NF-*κ*B signal activation. Furthermore, SIRT3 significantly inhibited IL-1*β*-induced NF-*κ*B signal activation in human FLS in vitro.

### 3.5. SIRT3 May Regulate NF-*κ*B Expression in Human FLS

**Figure 5:**
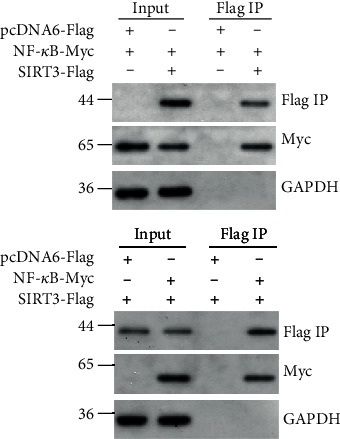
The endogenous coimmunoprecipitation in human FLS. Recombinant plasmids (SIRT3-pcDNA6-Flag and NF-*κ*B-pcDNA6-Myc) were transferred into FLS. Endogenous coimmunoprecipitation was performed in human FLS.

The regulation mechanism of SIRT3 in the NF-*κ*B signal pathway was further investigated. Recombinant plasmids (SIRT3-pcDNA6-Flag and NF-*κ*B-pcDNA6-Myc) were transferred into FLS. Endogenous coimmunoprecipitation was performed and showed that SIRT3 may directly bind with NF-*κ*B (Figure 5).

## 4. Discussion

OA is a prevalent joint disease primarily characterized by joint pain and articular cartilage degradation. In recent years, an increasing number of studies have focused on the contribution of synovial inflammation to the pathology of OA [17, 18]. Several studies have shown that more than 50% of OA patients suffer from synovitis [19], and synovial inflammation is strongly associated with the rapid progression of cartilage loss in OA [20]. Synovial inflammation is characterized by synovial lining hyperplasia, stromal vascularization, and sublining fibrosis [21]. FLS, the primary cells in the synovial membrane, are actively involved in chronic inflammatory reactions. During OA, FLS proliferate rapidly and attach to the articular cartilage. They produce chemokines and cytokines, such as IL-1*β*, THF-*α*, and IL-6, to attract other immunes cells around the articular cartilage and induce chondrocytes to secrete cartilage-degrading enzymes and protease, such as MMPs [22–24]. Oxidative stress and ROS have been regarded as major factors that lead to the onset and progression of OA [25]. Oxidative stress promotes peroxiredoxin (PRX) hyperoxidation, which disrupts normal physiological signaling and contributes to OA [26]. During OA progression, various inflammatory mediators, such as IL-1*β* and INOs, increase ROS accumulation and oxidative stress, which causes mitochondrial and nuclear DNA damage and epigenetic changes in gene expression that contribute to the progression of OA [23, 27]. ROS have a distinct contribution to oxidative stress in synovial inflammation [28]. ROS induce COX2 overexpression and oxidative stress activation. This affects cell apoptosis and impairs the balance between anabolism and catabolism in FLS [29–31]. During synovial inflammation, FLS additionally produce the receptor activator of nuclear factor kappa-B ligand (RANKL), which activates osteoclasts and leads to loss of bone mass [32, 33]. Many studies have shown that IL-1*β* can break the homeostasis of FLS due to the upregulation of inflammatory cytokines and matrix-degrading genes, such as MMPs. In this study, FLS were incubated with or without IL-1*β* (10 ng/ml) for 24 h in vitro. The resulting data showed that the expressions of inflammatory cytokines (COX2, PGE2, ADAMTS4, iNOS6), MMP3, and ROS were increased by IL-1*β* in FLS.

The primary active component derived from *Fructus Ligustri Lucidi*, OLA, has been reported to ameliorate chondrocyte inflammation [14]. For the first time, to the best of our knowledge, we reported the anti-inflammatory properties of OLA in OA synovitis. Our data showed that OLA increased the proliferation activity of FLS and significantly decreased the expression of COX2, PGE2, ADAMTS4, iNOS6, and MMP3 induced by IL-1*β* in human FLS.

OLA also significantly reduced the high levels of ROS induced by IL-1*β* in FLS. Furthermore, the potential mechanism of the anti-inflammatory result of OLA was explored in that study, and it was found OLA significantly inhibited phosphorylation and acetylation of NF-*κ*B. These results demonstrated that OLA showed a therapeutic potential in OA synovitis.

More importantly, it was found in the present study that OLA significantly increased the expression of SIRT3 in FLS. It was further discovered that SIRT3 was expressed in the synovial membrane of OA. These findings suggested that SIRT3 may play an important role in pathological process of OA synovitis.

Mitochondrial dysfunction during aging has been confirmed in chondrocytes and is associated with OA [34–36]. Sirtuins, a family of nicotinamide adenine dinucleotide- (NAD-) dependent histone deacetylases, deacetylate protein targets and regulate cell physiological and pathological processes. An increasing number of studies have shown that SIRT3, a member of Sirtuins family located in mitochondria, acts as a “master” regulator of mitochondria in chondrocytes. During OA, the level of SIRT3 is reduced in chondrocytes. SIRT3 knockdown induces mitochondrial dysfunction and inflammation due to apoptosis and autophagy in chondrocytes [37–40]. However, there are no existing studies regarding the regulation of SIRT3 in OA synovitis. This study showed that SIRT3 was expressed in OA synovial tissues. Then, it was found that the overexpression of SIRT3 significantly decreased the IL-1*β*-induced upregulation of inflammatory genes (COX2, PGE2, ADAMTS4, and iNOS6) and MMP3 in human FLS. In contrast, knockdown SIRT3 significantly increased the IL-1*β*-induced upregulation of inflammatory markers. The overexpression SIRT3 also significantly reduced the high level of ROS induced by IL-1*β* in FLS.

There is still a question regarding how SIRT3 decreases inflammation and oxidative stress in OA synovitis. Some studies have shown that the SIRT3 promoter can be activated by the stress transcription factor NF-*κ*B, and this resulted in activation of the SIRT3 transcriptional in human tumor cells [41–43]. The NF-*κ*B signal pathway is well recognized to participate in the progress of OA [44–46]. In this study, it was hypothesized that SIRT3 may regulate synovial inflammation via NF-*κ*B activation. The data suggested that reduced SIRT3 expression is associated with elevated NF-*κ*B phosphorylation and acetylation. SIRT3 inhibited the phosphorylation and acetylation of NF-*κ*B. Furthermore, it was found that SIRT3 may directly bind with NF-*κ*B in FLS. However, further studies are needed to elucidate the exact mechanism by which SIRT3 regulates the NF-*κ*B signal pathway.

## 5. Conclusion

In this study, it was found that OLA inhibited synovial inflammation and increased SIRT3 expression in FLS. SIRT3 inhibited FLS inflammation in vitro via suppression of the NF-*κ*B signal pathway. It is proposed that OLA has therapeutic potential in OA synovitis, and the targeting of SIRT3 may be a potential therapeutic treatment to combat OA synovitis.

## Figures and Tables

**Table 1:** Primer sequences used in this study.

Gene	GenBank accession	Primer sequences (5′ to 3′)	Size (bp)	Annealing (°C)
Human GAPDH	NM_002046.5	CCATGACAACTTTGGTATCGTGGAA	107	60
		GGCCATCACGCCACAGTTTC		
Human MMP3	NM_002422.5	CCTCCAACCGTGAGGAAAATCG	153	60
		CCCTGGAAAGTCTTCAGCTATTTG		
Human ADAMTS4	NM_005099.6	CCTCAATGGTGAATACACGCTGATG	153	60
		GCCACTAGGACTTGCAGTGTCAAA		
Human iNOS	L09210	CCGAGGCAAACAGCACATTCAGAT	87	60
		GAGTCCTGCACGAGCCTGTA		
Human SIRT3	NM_012239.6	GCGGCTCTACACGCAGAACATC	100	60
		GCAGGTGGCAGAGGCAAAGGT		

## Data Availability

The datasets generated for this study are available upon request to the corresponding author.
